# Absence of Vitamin D Deficiency Among Outdoor Workers With Type 2 Diabetes Mellitus in Southern West Bengal, India

**DOI:** 10.7759/cureus.22107

**Published:** 2022-02-10

**Authors:** Soumik Goswami, Neha Agrawal, Nilanjan Sengupta, Arjun Baidya, Pranab K Sahana

**Affiliations:** 1 Endocrinology, Nilratan Sircar Medical College, Kolkata, IND

**Keywords:** vitamin d deficiency, type 2 diabetes mellitus, outdoor workers

## Abstract

Background: Vitamin D deficiency is widespread globally and is associated with type 2 diabetes mellitus (T2DM). Studies suggest markedly lower prevalence of vitamin D deficiency in outdoor workers compared to indoor workers. However, data on the vitamin D status of outdoor workers with T2DM is lacking.

Aims: We assessed the vitamin D status of outdoor workers with T2DM residing across several districts of Southern West Bengal, India.

Design: The present study is a descriptive observational study.

Material and methods: A total of 128 outdoor workers with T2DM were assessed for serum 25-hydroxyvitamin D (25(OH)D) during December 2019 after excluding common confounders except sun exposure (which was detailed using a questionnaire). Hospital staff were indoor controls, and vitamin D status was classified as per the Institute of Medicine guidelines.

Results: The mean serum 25(OH)D of outdoor workers with T2DM was 21.79 ± 6.31 ng/mL, with only 2.34% (n = 3) having vitamin D deficiency and 57.03% (n = 73) having sufficient serum 25(OH)D levels. The mean serum 25(OH)D of indoor controls was significantly lower at 16.67 ± 9.82 ng/mL (p = 0.003), with 33.33% being vitamin D deficient. Serum 25(OH)D in outdoor workers with T2DM did not have a significant correlation with indices of sun exposure.

Conclusions: Vitamin D deficiency is practically absent in outdoor workers with T2DM residing in Southern West Bengal, India.

## Introduction

Vitamin D, the “sunshine vitamin,” plays an important role in maintaining bone health and has a possible role in several extraskeletal disorders including type 2 diabetes mellitus (T2DM) [1-3]. Although meta-analyses do not show significant effects of vitamin D supplementation on glycemic control, Indian studies have shown its benefit in improving metabolic parameters in prediabetes patients [1,3]. Vitamin D supplementation also exerts a salutary effect on skeletal disorders, which are widely prevalent in T2DM [4]. Generally, vitamin D status is assessed by serum 25-hydroxyvitamin D (25(OH)D) levels, with approximately 85%-90% of 25(OH)D circulating in association with vitamin D-binding protein [5]. Vitamin D studies have shown its deficiency to be a major health problem affecting an estimated one billion individuals globally, being prevalent not only in countries with cold climates but also in sunny regions such as India [6,7]. Dark skin complexion, limited sun exposure, using umbrellas and hats, using sunscreen, staying in the shade, and wearing full-body covering clothes are common in India, which contribute to vitamin D deficiency [8]. Indian studies have shown a very high prevalence of vitamin D deficiency in patients with T2DM (30%-85%) [9,10]. However, these studies have primarily looked at urban populations and not at outdoor workers separately who might be vitamin D sufficient due to adequate occupation-driven sun exposure [11-13]. A systemic review in 2017 clearly showed a higher level of serum 25(OH)D in outdoor workers compared to indoor workers [14]. From an Indian perspective, this assumes special significance as about 75% of its total labor force is engaged in outdoor work, including farming and construction activity [15]. There are two studies from North India that looked at outdoor workers in New Delhi and found a considerably low prevalence of vitamin D deficiency [16,17]. A small study from South India also found normal serum 25(OH)D levels in outdoor laborers and fishermen from Thiruvananthapuram [18]. However, these studies did not exclusively study outdoor workers with T2DM, and there is no published study from Eastern India looking at outdoor workers, which is important as this part of the country lies at a different latitude from New Delhi and Thiruvananthapuram. Assessing the vitamin D status of people working outdoors with T2DM will help design strategies for testing and supplementation of vitamin D. The aim of this study was to assess the vitamin D status of outdoor workers with T2DM residing across several districts of Southern West Bengal, India.

## Materials and methods

The study was carried out in the month of December 2019 among outdoor workers with T2DM attending the diabetes outpatient department (OPD) of a tertiary care hospital in Kolkata. The details of the criteria used for selecting the study population are mentioned in Table 1. Hospital staff (faculty doctors, postdoctoral students, nurses, and dieticians) were taken as controls as they were employed in indoor work from 9:00 AM to 4:00 PM. Hospital staff were chosen as indoor controls on the account of the convenience of recruitment and blood sampling. All blood samples were collected in the month of December to avoid seasonal variations in serum 25(OH)D levels.

**Table 1:** Inclusion and exclusion criteria for study population selection

Inclusion criteria	Exclusion criteria
Individuals with T2DM working for at least the last six months in their respective outdoor jobs	Point of care capillary blood glucose ≥ 200 mg/dL
HbA1c ≤ 7.5% [19]	Use of vitamin D supplements during the last six months
eGFR > 60 mL/minute/1.73 m^2^ [20]	Use of sunscreen during the last six months

All outdoor workers with T2DM were interviewed individually about the details of their district of residence, occupation, work schedule, clothes worn, and pattern of daily sunlight exposure using a questionnaire. Body surface area exposed to sunshine was calculated by the rule of nine [21]. Sun index was calculated as the product of sunshine exposure in hours/week and the fraction of body surface area exposed [22].

Blood (5 mL) was drawn from both groups for assaying serum 25(OH)D by chemiluminescence assay using Beckman Coulter Access 2 (Beckman Coulter Inc., Brea, California, USA), which has a total imprecision of ≤10% coefficient of variation (CV) at concentrations of >15 ng/mL (37.5 nmol/L) and standard deviation (SD) of ≤1.5 ng/mL (3.8 nmol/L) at concentrations of ≤15 ng/mL. Vitamin D status was defined in accordance with the report of the Institute of Medicine committee, with serum 25(OH)D ≥ 20 ng/mL considered as sufficient, 12-20 ng/mL as insufficient, and <12 ng/mL as deficient [23].

The study was carried out in sync with the tenets of the Code of Ethics of the World Medical Association (Declaration of Helsinki), and written informed consent was obtained from all participants.

The sample size was based on the vitamin D status of outdoor workers in a previous study from New Delhi, India, which showed a 14.8% prevalence of serum 25(OH)D < 20 ng/mL in outdoor workers [16]. Assuming a similar vitamin D status among the outdoor workers of the present study, a total of 103 participants were required to estimate the prevalence of vitamin D deficiency with an assumed sensitivity and specificity of 90% with 90%, respectively, in a two‐sided 95% confidence interval. Student’s t-test was used to compare serum 25(OH)D levels between outdoor population and indoor controls, and Pearson’s correlation coefficient was used to analyze the association between serum 25(OH)D levels in the outdoor group and the indices of sunlight exposure. Data were tabulated and analyzed using Microsoft Excel 2019 (Microsoft Corp., Redmond, WA, USA).

## Results

A total of 128 outdoor workers with T2DM were recruited in the study, of whom the majority were males (n = 122, 95.3%). The mean age of the outdoor workers was 50.1 ± 9.62 years. The outdoor workers were from seven districts of Southern West Bengal - North 24 Parganas (n = 41, 32.03%), Nadia (n = 28, 21.88%), South 24 Parganas (n = 27, 21.09%), Murshidabad (n = 13, 10.16%), Kolkata (n = 10, 7.81%), Hooghly (n = 5, 3.91%), and Howrah (n = 4, 3.13%). In terms of occupation pursued, the majority were expectedly engaged in farming (n = 76, 59.38%). Other occupations included masons (n = 21, 16.41%), miscellaneous outdoor work (n = 20, 15.63%), hawkers (n = 7, 5.47%), and fishermen (n = 4, 3.13%). The total daily sun exposure and 10:00 AM to 3:00 PM sun exposure were quite high at 7.18 ± 2.31 hours and 3.27 ± 1.51 hours, respectively. The body surface area exposed was 29.8% ± 2.67%, and the sun index was 13.52 ± 8.37. A total of 42 hospital staff were included as indoor controls, with a mean age of 30.90 ± 10.20 years, with 22 of them being males.

The mean serum 25(OH)D level of outdoor workers with T2DM was 21.79 ± 6.31 ng/mL; 57.03% (n = 73) of them had sufficient serum 25(OH)D levels, 40.63% (n = 52) had vitamin D insufficiency, and only 2.34% (n = 3) had vitamin D deficiency (Table 2). The mean serum 25(OH)D level in the outdoor group was significantly higher than that of the indoor controls (16.67 ± 9.82 ng/mL, p = 0.003) (Table 2). The serum 25(OH)D levels of outdoor workers with T2DM did not have a significant relationship with total sunlight exposure, 10:00 AM to 3:00 PM sunlight exposure, body surface area exposed, and sun index in our study population (p > 0.05).

**Table 2:** Serum 25(OH)D levels in the study population

Parameters	Outdoor	Indoor	p
Vitamin D level (ng/mL)	21.79 ± 6.31	16.67 ± 9.82	0.003
Serum 25(OH)D ≥ 20 ng/mL (vitamin D sufficient)	73 (57.03%)	8 (19.05%)	
Serum 25(OH)D between 12 and 19.99 ng/mL (vitamin D insufficient)	52 (40.63%)	20 (47.62%)	
Serum 25(OH)D < 12 ng/mL (vitamin D deficient)	3 (2.34%)	14 (33.33%)	

## Discussion

Vitamin D deficiency has assumed alarming prevalence globally, which has elicited significant research interest to elucidate its potential causes in order to advance appropriate interventional strategies [6,24]. Several studies from India and abroad have shown widely prevalent vitamin D deficiency in patients with T2DM [3,9,10]. On the other hand, various studies have shown that outdoor workers have adequate sunlight exposure, and most of them usually have sufficient serum 25(OH)D levels [14,16,17]. The present study was carried out to assess the vitamin D status of outdoor workers with T2DM - an area where there is a dearth of data not only from India but also globally.

Our study showed optimal serum 25(OH)D levels (21.79 ± 6.31 ng/mL) among outdoor workers with T2DM, with values ranging from 11.4 ng/mL to 45.23 ng/mL, with the duration of daily exposure to sunshine being 7.18 ± 2.31 hours. The three Indian studies published earlier that looked at serum 25(OH)D in outdoor workers are shown in Table 3. Our study was conducted in December, while the outdoor workers in the study of Goswami et al. were studied between August and October and had a greater duration of daily sun exposure of 9.0 ± 0.0 hours [16]. The study of Dharmshaktu et al. had a good number of autorickshaw drivers included as outdoor workers who were mostly exposed to indirect sunlight and had lower serum 25(OH)D levels (14.56 ± 7.16 ng/mL) than the overall study population, which could be a reason for this study showing relatively lower overall serum 25(OH)D levels [17]. The study of Rajasree et al. from South India analyzed data from only 37 outdoor workers who worked in a coastal environment within 12° of the equator and received abundant sunshine throughout the year [18]. Sharan et al. found a mean serum 25(OH)D level of 24.91 ± 14.58 ng/mL among 120 newly diagnosed patients with T2DM in Kolkata, although these individuals were not grouped according to outdoor or indoor occupational activity [9]. None of these studies from India exclusively looked at outdoor workers with T2DM as was done in our study.

**Table 3:** Indian studies looking at serum 25(OH)D in outdoor workers

Study	Year, place	Mean serum 25(OH)D in ng/mL
Rajasree et al. [18]	1999, Thiruvananthapuram	169.8 (laborers), 110.2 (fishermen)
Goswami et al. [16]	2017, New Delhi	29.0 ± 8.61
Dharmshaktu et al. [17]	2019, New Delhi	17.92 ± 7.84
Present study	December 2019, Kolkata	21.79 ± 6.31

Vitamin D deficiency was practically absent (about 2%) among outdoor workers with T2DM in our study; the majority of about 57% had sufficient serum 25(OH)D levels, while about 41% had vitamin D insufficiency. The study of Goswami et al. similarly showed an absence of vitamin D deficiency in outdoor workers of New Delhi, although the prevalence of vitamin D sufficiency was notably higher (about 85%) compared to our study [16]. In the study of Dharmshaktu et al., the prevalence of vitamin D deficiency was much higher (about 23%), while vitamin D sufficiency was lower (about 34%) compared to our study [17]. Sharan et al. found that about 13% of the newly diagnosed patients with T2DM at Kolkata had a vitamin deficiency, while about 58% had sufficient serum 25(OH)D levels [9].

In our study, the outdoor group expectedly had significantly higher serum 25(OH)D levels than the indoor controls (16.67 ± 9.82 ng/mL, p = 0.003), in tune with the findings of a systematic review of 71 peer-reviewed articles [14]. Hospital staff were included as indoor controls on the account of the associated convenience of recruitment with the known limitation that they did not have T2DM. However, since this was not a primary objective of our study and since those without T2DM are expected to have similar or higher serum 25(OH)D levels than those with T2DM, the interpretation of results remains unchanged [9].

In our study, there was no significant association between serum 25(OH)D levels in outdoor workers with T2DM and either total sunlight exposure, 10:00 AM to 3:00 PM sunlight exposure, body surface area exposed, or sun index. A study from Pune by Patwardhan et al. found a hyperbolic relationship between sunlight exposure and serum 25(OH)D levels: >1 hour of casual midday sunlight exposure daily maintained serum 25(OH)D concentrations above 20 ng/mL, and >2 hours of casual sunlight exposure maintained 25(OH)D concentrations above 30 ng/mL, with excess sunlight not increasing 25(OH)D linearly [25]. In our study, the sufficient duration of both 10:00 AM to 3:00 PM sun exposure and total daily sun at 3.27 ± 1.51 hours and 7.18 ± 2.31 hours, respectively, and sufficient serum 25(OH)D levels in the majority of outdoor workers with T2DM could possibly explain the lack of an association between sun exposure and serum 25(OH)D levels.

The strengths of our study include the inclusion of a racially homogenous group belonging to lower-middle socioeconomic status recruited over a very short time span to limit the seasonal variation in serum 25(OH)D. The present study has some limitations: inadequate representation of female outdoor workers, lack of documentation of dietary vitamin D intake, the fabric of clothes, minor differences in skin complexion, and time spent in the shade during outdoor work. Another limitation of our study as discussed earlier is the unmatched control group. However, these limitations are unlikely to change the interpretation of the data in practical terms.

The present study suggests that a large section of the population of Southern West Bengal with T2DM who are engaged in outdoor activities with prolonged sun exposure might have sufficient serum 25(OH)D levels, and in them, unnecessary testing for serum 25(OH)D and vitamin D supplementation can be avoided.

## Conclusions

Our study shows the near absence of vitamin D deficiency in outdoor workers with T2DM in Southern West Bengal, India, with the majority of them instead having sufficient serum 25(OH)D levels.
